# Supplementary material for: Alcohol, tobacco and drug use among adults experiencing homelessness in Accra, Ghana: A cross-sectional study of risk levels and associated factors
Source: PLoS One. 2023 Mar 6;18(3):e0281107. doi: 10.1371/journal.pone.0281107 (PMC9987824; doi:10.1371/journal.pone.0281107)
Supplement: S1 Table — (DOCX) [file pone.0281107.s001.docx]

**Interaction between Gender and Violence for Overall High-risk Substance Use**

**Interaction between Gender and Violence for Overall High-risk Alcohol use**

**Interaction between Gender and Violence for Overall High-risk Cocaine Use**

**Interaction between Gender and Violence for Overall High-risk Cannabis Use**

**Interaction between the Different types of violence**
