# Supplementary material for: Alcohol, tobacco and drug use among adults experiencing homelessness in Accra, Ghana: A cross-sectional study of risk levels and associated factors
Source: PLoS One. 2023 Mar 6;18(3):e0281107. doi: 10.1371/journal.pone.0281107 (PMC9987824; doi:10.1371/journal.pone.0281107)
Supplement: Attachment — Submitted filename: Response to Reviewers Comments.docx [file pone.0281107.s003.docx]

**RESPONSE TO REVIEWERS’ COMMENTS**

| **Reviewer** | **Comment** | **Response** |
| --- | --- | --- |
| 1 | Line 42: Please rephrase the sentence “The likelihood of engaging in high-risk substance used higher for men than women 43 (AOR=4.09; 95%CI 2.06-8.12, p<.001)” Did authors mean “the likelihood of engaging in high-risk substance use was higher for men than women? | The abstract has been revised to incorporate the suggested change. Yes, we have changed the statement to “the likelihood of engaging in high-risk substance use was higher for men than women” |
|  | -There are terms introduced in the text starting from the abstract that may require inclusion of their standard definition in the method section: What do the authors mean by “sheltered and unsheltered homelessness” | Thank you for the comment and suggestions. We have defined experiences of sheltered and unsheltered homelessness in the methods (section 2.2: Study respondents) |
|  | Line 112: “Since there was no official data on homelessness in Ghana, the uncontrolled quota sampling technique [34, 35] was used to obtain a representation of sheltered and unsheltered homeless populations” -Please add justification for using uncontrolled quota sampling, how uncontrolled was the sampling procedure? | Thank you for the suggestions. We have explained and justified our use of the quota sample techniques |
|  | Line 157: Substance use risk levels were assessed using the ASSIST version 3.0, a tool to identify levels of risky substance use and related health risks -For those who used local language, is there a translated validated ASSIST tool as per the local language? please specify, it will be great to also report the validity and reliability of the translated tool | Thank you for the comment and suggestion. The manuscript has been amended to explain the procedure used to translate the ASSIST instrument into the local language to accommodate participants who were unable to speak the English Language |
|  | Line 183: Experiences of stigmatisation: Assessed using a 12-item Social Stigma Scale to measure the experience of general stigma -Same here, please report on the internal consistency and validity of the tool used | Thank you for the comment and suggestion. We have provided information on the reported validity and reliability of the Social Stigma Scale and its prior use in Ghana in the revised version. We have also explained the translation of the instrument into the local language to accommodate participants who were unable to speak the English Language |
|  | Line 192: Violence and abuse: measured using items from the Ghana Living Standard Survey (GLSS) Round 6  - Same here, please report on the internal consistency and validity of the tool used | Thank you for the comment and suggestion. The manuscript has been amended to explain the procedure used to translate the violence and abuse items were into the local language to accommodate participants who were unable to speak the English Language |
|  | -Please specify the level of statistical significance used | The data analysis section has been amended to specify the level of statistical significance (p≤.050) |
|  | Line 188: Self-reported experience or diagnosis of a range of physical and mental health conditions in the past three months -Weren’t the patients who reported mental health conditions diagnosed in the past three months excluded? This was one of the exclusion criteria reported in the study participant section “individuals with cognitive impairment, intellectual disability, and mental illness were excluded from participation following advice from social workers and shelter staff.” Please clarify how comes those with mental health diagnosis were included. | Thank you for the comment.  What we meant was that people with mental health problems were excluded based on the seriousness of the condition and their capacity to provide consent. That is individuals with serious cognitive impairment, intellectual disability, and mental illness were excluded if the conditions limited capacity to consent. For such individuals, the capacity to provide consent was determined by social workers and shelter staff based on the seriousness of the health condition and prior experience of serious distress. We have revised the methods section to well-explain this. |
|  | Table 2: Please specify, if possible, in bracket that the “Highest Specific Substance Index” has been reported as the overall risk to make it more clear | The results section has been revised to incorporate your suggestion-Line 309 |
|  | Line 270: “Moderate/high-risk substance users were also more likely have experienced general stigma than not (271 72% vs 51%, p<.001)” -add “to” have experienced | The results section has been revised to incorporate your suggestion-Line 300 |
|  | Line 342: “Although generally high, differences in prevalence exist between the present study and previous research” -While comparing the magnitude of risky substance use in literature, authors should take note of the differences in the substance use assessment tools used in other studies and how they can be comparable/correlated with ASSIST tool. | Thank you for the comment and suggestion. In comparing the current paper’s findings to those in the literature, we have highlighted the differences in problematic substance use assessment tools used in other studies. |
|  | Authors should highlight the disadvantages/ limitations that result from using uncontrolled quota sampling, biases resulting from non-randomization among others and how these were dealt with in the study | The non-random nature of the uncontrolled quota sampling has been highlighted under the study limitations. |
|  | With interview-based questionnaire, there is a risk of information bias, provision of socially desirable answers, especially since the use of some of the drugs is illegal in most African countries. These too are usually anticipated in studies relating to substance use hence should be reported if relevant. | Thank you for the comment and suggestion. We have acknowledged the possible risk of information bias and provision of socially desirable answers in the limitation section. However, the participants in the study openly talked about their substance use experiences including the perceptions on possible health impacts. |
|  | Please also comment on generalizability of the study findings | Thank you for the suggestion. We have commented on the generalisability of the study findings in the paragraph that discusses the study’s limitations. |
| 2 | Line 42: missing words… substance use were higher | The abstract has been revised to include the missing word |
|  | Line 46: Missing word… with the experience | The abstract has been revised to include the missing word |
|  | Line 86: Could some additional information iro of this larger cross sectional study be given and where this piece of work fits in or even provide a reference to a paper possibly published on the larger study. | The methods section has been revised to include additional information in the larger cross-sectional study. |
|  | Line 144: What would re-negotiate consent entail, please just expand on this and under what circumstance such a situation is likely to occur. | Thank you for the comment and suggestion. We have revised the procedure section to include explanation of consent re-negotiation. That is participants who had already agreed to participate were asked of permission again prior to the start of the survey. Participants could change their decision to participate or withdraw their participation altogether based on changes in personal lives or availability of new information about the research. |
|  | Line 146: Did the fieldworker pose the question to the participant and enter it on behalf of the participant or were the questions posed and the participant themselves entered the response on the tablet. Possibility of bias which should be detailed in the limitations if not there. | The fieldworker posed the question to the participant and entered it on behalf of the participant. We have included the possibility of bias in the limitation. |
|  | Line 151: Were referrals to centres facilitated by the field workers? Were the participants all given the option to do this or was it based on a score on the ASSIST? | Referrals were facilitated by the social workers and staffs at the rehabilitation centres. All participants had the option to check in. |
|  | Line 238: Perhaps added into the methods section under the relevant sub-heading, the authors should indicate what baseline was used to determine chronic homelessness versus “non-chronic” homelessness. | Thank you for suggestion. We have revised the ‘*Migration and homelessness characteristics’* part of under the measures to indicate the baseline used to determine chronic homelessness (≥1 year total lifetime duration of homelessness) versus “non-chronic” homelessness (<1 year total lifetime duration of homelessness) |
|  | Line 244: Presumably data on whether participants who self-reported an ailment sought help for physical/mental health problems was not collected? If yes it would be interesting to know. | Data on health care utilisation by the study participants including were collected. However, that was not the focus of this paper. There is a whole chapter of the larger cross-sectional study that is dedicated to health care utilisation, where we have presented data on what proportion of the participants who reported health problem (physical & mental) utilised health care, and what type, if utilised. |
|  | Line 271: more likely to have experience… missing word | The section has been revised to include the missing word |
|  | Lines 259-363 – Is there are reference for this, because there may be a divergent view iro of the value/effectiveness on measured outcomes of outpatient treatment vs inpatient – there is evidence of the value of outpatient/community-based services to. Perhaps the authors should do a quick review to look into this and provide references. This would apply to line 364 to mid-paragraph. | Thank you for the comment and suggestions. We have included references to support the effectiveness of the inpatient rehabilitation services for people who experience homelessness. |
