# Supplementary material for: Alcohol, tobacco and drug use among adults experiencing homelessness in Accra, Ghana: A cross-sectional study of risk levels and associated factors
Source: PLoS One. 2023 Mar 6;18(3):e0281107. doi: 10.1371/journal.pone.0281107 (PMC9987824; doi:10.1371/journal.pone.0281107)
Supplement: Attachment — Submitted filename: RESPON~1.DOC [file pone.0281107.s004.DOC]

**RESPONSE TO REVIEWERS’ COMMENTS**

| **Reviewer** | **Comment** | **Response** |
| --- | --- | --- |
| 1 | Line 131-133: Refer to “The initial plan was to sample 70% unsheltered and 30% sheltered homeless persons, and the final sample consisted of 68% unsheltered and 32% sheltered persons.” Please move this statement that explains on the quota weightage just after mentioning quota sampling method. | Thank you for the comments, we have revised methods section to reflect the suggested changes (Line 124-129). |
|  | Line 125-127: The authors report the sample size to be deemed sufficient to determine predictors. If possible, I would suggest reporting the power of the study at 5% significance level to support this. | Thank you for this comment. The authors acknowledge the importance of calculating and reporting the statistical power of a study. However, we are unable to calculate the statistical power during the design phase of this study due to absence of information on the homeless population in Ghana (population size & characteristics). Hence, the sample size of 305 (which is actually larger than the planned 200) is deemed sufficient for our purposes, which did not include hypotheses testing. Also, our analyses were adjusted to account for low frequency responses and effect sizes and confidence intervals were reported for all analyses undertaken. |
|  | Line 157: Substance use risk levels were assessed using the ASSIST version 3.0, a tool to identify levels of risky substance use and related health risks –  Line 173: This is a follow up from the previous comment: For those who used local language, is there a translated validated ASSIST tool as per the local language in Ghana? it will be great to also report the validity and reliability of the translated tool, same applies to the GLSS  NB: Some tools have been pre-translated into different languages for use by different localities, some of which will have studies reporting the validity of such translated tools. Hence, this question asks about the translated ASSIST tool, not the language used for the questionnaire – refer to the revision made by the authors on the Stigma tool | We appreciate your comments. The authors did a careful selection of the various instruments used in the study. Since its introduction in Ghana in February 2008 by the WHO Regional Office for Africa (WHO/AFRO) in consultation with Ghana’s Ministry of Health, few trained health workers have been using the ASSIST early detection of risky substance use in health settings. However, the instrument is not available in any Ghanaian local language, and health workers translate the items directly during administration.  Since this is the first and only Ghanaian study to use the ASSIST, we translated the instrument to better understanding and quality control, and also minimise the challenges associated with reading items written in English to participants in the Ghanaian language. The translation was conducted using the WHO translation guidelines quality assurance in surveys. We established the validity of the questionnaire by undertaking a detailed review of related literature on studies that have used the ASSIST instrument in sub-Saharan African countries like Nigeria with socio-demographic characteristics similar to Ghana’s. The section describing the ASSIST instrument has been revised to reflect this explanation (Line 187-190)  Items to assess violent victimisation were obtained the Ghana Living Standard Survey, which were already translated into nine Ghanaian languages, including the Twi language used in the survey. There is no reported validity of the GLSS questionnaires, but items for measuring violence in this study were selected based on extensive review of literature on the subject matter. |
|  | Line 298: Authors should be consistent with the statistical method used: is it univariate or bivariate? Please be consistent throughout. | We have revised the manuscript to reflect the suggested changes. We have used bivariate throughout the manuscript. |
|  | Line 320: Before reporting the AOR, authors may consider mentioning which variables were exclude in the multivariable model based on the p<0.25 cut-off as specified. | Thank you for the comment. We have revised the data analysis section to report all variables that were excluded from each of the multivariable regression models based on the p<0.25 cut-off (Line 253-265). |
|  | Line 340, table 4: Please clarify what the empty spaces in the table implies and if possible, indicate in the table legends. Indicate the long forms too in the legends for each table. | The empty spaces in the table 4 represents the variables that we excluded from the multivariable analysis based on the p<0.25 cut-off. |
|  | Line 340, table 4: Some factors that had p values >2.5 on bivariate analysis included in the multivariable model. For instance, the variable Mental health problems and self-blame had p values of 0.36 on bivariate analysis, yet, they were included in the multivariable model. Please clarify. NB: It is useful for the authors to mention the inclusion of factors that may be insignificant statistically but could be included in the model apriori, mental health problems is a good example here. | Self-blame (a sub-category of social stigma) was included apriori after extensive review of literature revealed that people may engage in problematic substance use as a coping strategy for stigmatisation including guilt and self-blame. Also, the qualitative part of the larger research project found that individuals experiencing homelessness (mostly women) engaged in substance use to cope with stigma and self-blame related to homelessness and experience of sexual assault.  Presence of mental health problems was included apriori due to established relationship with substance use in both the general and marginalised populations.  The manuscript has been revised to mention the inclusion of self-blame and mental health variables in the multivariable models apriori (Line 261-265)  It is useful for the authors to mention the inclusion of factors that may be insignificant statistically but could be included in the model apriori, mental health problems is a good example here. |
|  | Line 381-384: “Although generally high, differences in prevalence exist between the present study and previous research” -Mental health diagnosis varies as compared to other diseases, because of the presence of number of tools. While comparing the magnitude of risky substance use in literature, authors should take note of the differences in the substance use assessment tools used in other studies and how they can be comparable/correlated with ASSIST tool (Please indicate where changes were made) | We have compared the results of studies that used different substance use tool to this study’s results (Line 398-402) |
|  | Line 453-454: The authors mentioned the representativeness of the quota sampling used for this study, please do also outline the limitation of using non-random sampling.  I would suggest a notable response to this, from previous comments: Please also comment on “generalizability of the study findings” ie. taking into account the sample size, are the authors able to make broad inferences about the findings from this study?? | Thank you for the suggestion. We have included the limitations of using non-random sampling, including possible bias and limited generalisability (Line 474-477).  The small sample from a single city (Accra) also make also makes it difficult to make broad inferences about the findings from this study to the larger Ghanaian homeless population. We have included this in the manuscript (Line 477-479) |
