# Supplementary material for: Alcohol, tobacco and drug use among adults experiencing homelessness in Accra, Ghana: A cross-sectional study of risk levels and associated factors
Source: PLoS One. 2023 Mar 6;18(3):e0281107. doi: 10.1371/journal.pone.0281107 (PMC9987824; doi:10.1371/journal.pone.0281107)
Supplement: Attachment — Submitted filename: RESPON~1.DOC [file pone.0281107.s005.DOC]

**RESPONSE TO REVIEWERS’ COMMENTS**

| **Comment** | **Response** |
| --- | --- |
| Please review your reference list to ensure that it is complete and correct. If you have cited papers that have been retracted, please include the rationale for doing so in the manuscript text, or remove these references and replace them with relevant current references. Any changes to the reference list should be mentioned in the rebuttal letter that accompanies your revised manuscript. If you need to cite a retracted article, indicate the article’s retracted status in the References list and also include a citation and full reference for the retraction notice. | Thank you for the comment. We have reviewed the reference list to ensure its completeness and correctness. We have formatted the referencing to the Vancouver style in accordance to the journal’s requirements. The number of cited articles has been reduced from 112 in the previous version to 107. Five dated referenced were removed in favour of more recent articles. |
| Thanks for all the revisions. The paper reads well. Please kindly format the paper according to the journal format. | Thank you very much. The paper has been reformatted according to the journal’s format. We followed the checklist of items that should be included in reports of cross-sectional studies. |
