# Supplementary material for: Alcohol, tobacco and drug use among adults experiencing homelessness in Accra, Ghana: A cross-sectional study of risk levels and associated factors
Source: PLoS One. 2023 Mar 6;18(3):e0281107. doi: 10.1371/journal.pone.0281107 (PMC9987824; doi:10.1371/journal.pone.0281107)
Supplement: Attachment — Submitted filename: RESPON~1.DOC [file pone.0281107.s006.DOC]

**RESPONSE TO REVIEWERS’ COMMENTS**

| **Comment** | **Response** |
| --- | --- |
| Thanks for the revisions and the paper reads well. I have one critical comment - it would be helpful to standardise the reporting of  p values - decide whether to use 2 or 3 decimal points throughout the document. | Thank you very much. All p-values have been reported using three decimal points. |
